# Linked-In: Design and Efficacy of Antibody Drug Conjugates in Oncology

**DOI:** 10.18632/oncotarget.924

**Published:** 2013-03-26

**Authors:** Jonathan Feld, Stefan K. Barta, Carolina Schinke, Ira Braunschweig, Yiyu Zhou, Amit Verma

**Affiliations:** ^1^ Department of Medicine, Albert Einstein College of Medicine, Bronx, NY

**Keywords:** antibody drug conjugates, oncotargets, immunotoxins

## Abstract

The use of antibody drug conjugates (ADCs) as targeted chemotherapies has successfully entered clinical practice and holds great promise. ADCs consist of an antibody and toxin-drug combined together via a chemical linker. While the antibody and drug are of vital importance in the direct elimination of cancer cells, more advanced linker technology was instrumental in the delivery of more potent drugs with fewer side effects. Here, we discuss the preclinical experience as well as clinical trials, with a specific emphasis on the clinical outcomes and side effects, in addition to linker strategies for five different ADCs, in order to describe different approaches in the development of this new class of anticancer agents. Brentuximab vedotin is approved for use in Hodgkin&rsquo;s lymphoma and Trastuzumab emtansine is approved for breast cancer. Combotox, Inotuzumab Ozogamicin, and Moxetumomab Pasudotox are in various stages of clinical development and are showing significant efficacy in lymphoid malignancies. These ADCs illustrate the promise and future potential of targeted therapy for presently incurable malignancies.

## INTRODUCTION

**Figure 1:**
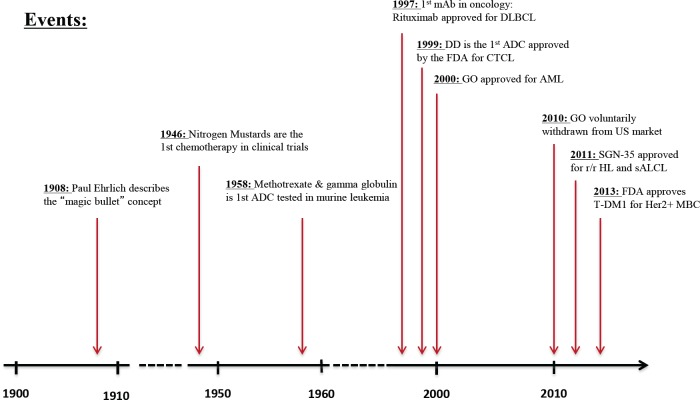
Timeline delineating the evolution of antibody-drug conjugate discovery and therapy in cancer therapeutics [3-6] ADC: Antibody-drug conjugate, mAb: Monoclonal antibody, DLBCL: Diffuse large B cell lymphoma, DD: Denileukin Difitox, CTCL: Cutaneous T cell lymphoma, GO: Gentuzumab Ozogamicin, AML: Acute myeloid leukemia, r/r: relapsed and/or refractory, HL: Hodgkin's lymphoma, sALCL: Systemic anaplastic large-cell lymphoma, FDA: Food and Drug Administration, Her2: Human Epidermal Growth Factor Receptor 2, MBC: Metastatic breast cancer.

The concept of linking an antibody to a toxin to create a safe and effective agent against cancer cells is not a new one. The magic bullet concept of Paul Ehrlich is over 100 years old,[1] while the first credible experiments linking chemotherapeutic agents to antibodies were performed almost 55 years ago).[2] At this point, despite all the years of research, there have only been four antibody drug conjugates (ADCs) approved by the FDA (Figure 1).[3-6] Brentuximab vedotin (SGN-35; Adcetris™) and Trastuzumab emtansine (T-DM1; Kadcycla™) were recently approved. Both revolutionized treatment for their respective indication (relapsed Hodgkin's lymphoma (HL)/systemic anaplastic large cell lymphoma (sALC), and Her2-positive breast cancer), while denileukin difitox (Ontak™) and, gemtuzumab ozogamicin (GO; Mylotarg™) have seen limited clinical use. In fact, GO was recently taken off the market in the United States.

**Figure 2:**
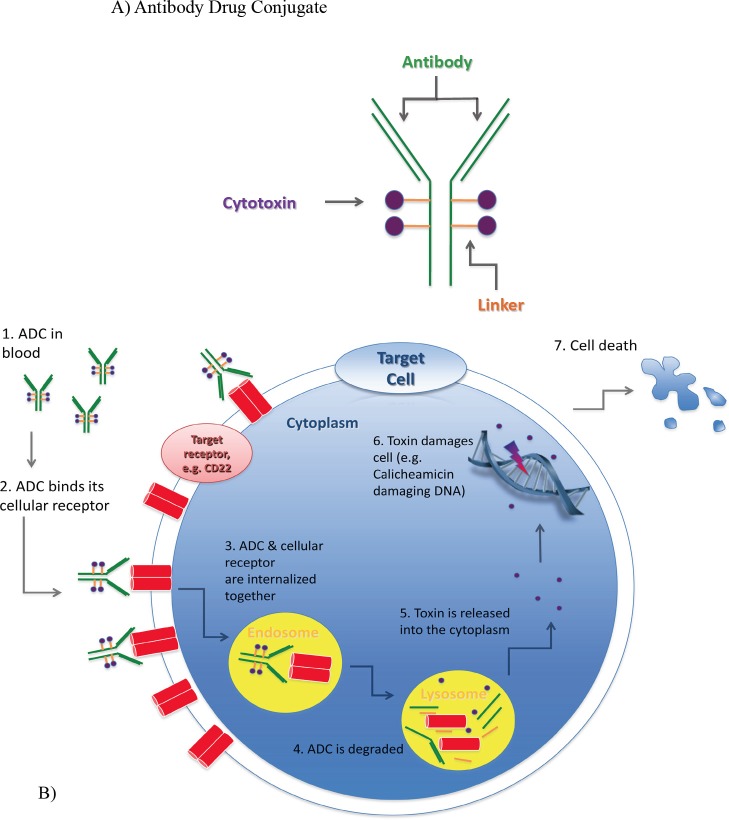
Composition and mode of action of antibody-drug-conjugates (ADC) A) An ADC is composed of a monoclonal antibody directed against a specific epitope on a target cell. A cytotoxic compound is attached to the antibody via a linker. B) Once administered, the antibody component of the ADC binds to the targeted cell receptor, which enables the ADC to be internalized (usually via endocytosis) and subsequently degraded. The released toxin causes cell death via various mechanisms depending on the toxin, such as DNA damage or inhibiting protein translation.

In general, ADCs comprise three components: they are made up of a monoclonal antibody (mAb) conjugated to a toxin via a chemical linker. The mAb allows targeted delivery of a potent cell toxin to specific malignant cells, thereby maximizing drug delivery while limiting bystander effects of traditional cytotoxic agents (Figure 2). In the last few years, it has become apparent that determining the perfect linker may be just as important as the other components of an ADC in increasing efficacy and decreasing toxicity.[7-9] This review describes in detail the development of five unique ADCs, each using a different combination of linker technology and toxin. These examples demonstrate that various components of an ADC are equally important in determining its efficacy and relative safety (Table 1).[10-13]

**Table 1:** Summary of the Antibody-Drug Conjugates

ADC	Brand Name	FDA approved	Target	Antibody	Linker	Toxin	Reference
**Combotox**	-	No	CD19 & CD22	RFB4 (CD22) & HD37 (CD19)	SMPT (disulfide)	dgRTA	[18]
**Moxetumomab Pasudotox**	-	No	CD22	Recombinant RFB4	C3 connector	PE38	[54]
**Inotuzumab Ozogamicin**	-	No	CD22	G5/44	AcBut (acid hydrolyzable)	CalichDMH	[81]
**Brentuximab Vedotin**	Adcetris™	Yes	CD30	cAC10	Valine-citrulline (dipeptide)	MMAE	[27]
**Trastuzumab emtansine**	Kadcycla™	Yes	Her2	Recombinant 4D5	MCC (thioether)	DM1	[37]

This is a summary overview of the five ADCs described in more detail in the text.

ADC: Antibody-drug conjugates, FDA: Food and Drug Administration, CD: cluster of differentiation, SMPT: *N*-succinimidyl-oxycarbonyl-a-methyl-a-(2-pyridyldithio)toluene, dgRTA: deglycosylated ricin-A chain, PE38: Pseudomonas Exotoxin a 38, AcBut: 4-(4'- acteylphenoxy)butanoic acid, CalichDMH: *N*-acetyl-gamma-calicheamicin dimethyl hydrazide, MMAE: monomethyl auristatin E, Her2: Human Epidermal Growth Factor Receptor 2, MCC: *N*-[maleimidomethyl] cyclohexane-1 carboxylate, DM1: maytasanoid N(2')-deacetyl-N(2')-(3- mercapto-1-oxopropyl)-maytansine

## DESIGN AND EFFICACY OF ANTIBODY DRUG CONJUGATES

### Combotox is a mixture of ricin-coupled antibodies against CD19 and CD22 and is effective in acute lymphoblastic leukemia

The immunotoxin (IT) Combotox is the 1:1 mixture of two murine monoclonal IgG1 antibodies, both individually linked to a ricin based toxin. It consists of RFB4, a CD22 antibody, and HD37, a CD19 antibody. Both antibodies are coupled to the toxin deglycosylated ricin-A chain (dgRTA), via *N*-succinimidyl-oxycarbonyl-a-methyl-a-(2-pyridyldithio)toluene (SMPT), a heterobifunctional, thiol-containing cross-linker. The toxin, dgRTA, is the isolated A-chain of the ricin toxic protein, which mediates its activity by disrupting the 60s ribosomal subunit, thereby inhibiting protein translation. It was later deglycosylated to reduce its hepatotoxicity.[14] CD22 is a B-cell specific antigen involved in regulating B-cell survival and function. It is an attractive target, in that it is expressed on the majority of B-cell cancers, but not by stem cell precursors (Table 2).[15] CD19 is also a B-cell specific antigen involved in cell signaling. It is expressed in many B-cell malignancies and is downregulated in plasma cells and hematopoetic stem cells, making it a similarly appealing target.[16] It is interesting to note that while there are generally more CD19-positive cancerous B-cells than CD22-positive cells, RFB4-dgRTA is the more potent IT. This could perhaps be explained by the far greater rate of CD22 internalization after RFB4 binding, compared to that of CD19 after HD37 binding.[17]

**Table 2:** Select targets of antibody-drug conjugates (ADC)

Target	ADC	Target Receptor Expression		Normal Function of Target Receptor	Reference
		Normal tissue	Malignant tissue		
**CD19**	Combotox	B cells, follicular DCs	ALL, CLL, NHL	B cell activation & signaling	[11]
**CD22**	Combotox, CMC- 544, HA22	Normal Pre-B & resting B cells	ALL, NHL, HCL, CLL	Regulates B cell survival & function	[12]
**CD25**	Denileukin Difitox	Activated T cells, B cells, & monocytes	CLL, ATL, CTCL, HCL, T-ALL	IL-2 receptor α chain-cell activation	[16]
**CD30**	SGN-35	Activated T, B, and NK cells, monocytes	HL, ALCL, lymphomas, embryonal carcinoma	Enhances B and T cell proliferation	[26]
**CD33**	Gemtuzumab ozogamicin	Myeloid progenitor cells, basophils, macrophages, DCs, monocytes	AML	Binds sialoconjugates. Regulates innate immunity & inflammation	[10]
**Her2**	T-DM1	Wide distribution (not hematopoietic cells)	Breast cancer, gastric cancer etc.	Assists in the activation of other EGFR proteins	[13]

CD: cluster of differentiation, ADC: Antibody-drug conjugates, DC: Dendritic cell, ALL: Acute lymphoblastic leukemia, CLL: Chronic lymphoblastic leukemia, NHL: Non-Hodgkin lymphoma, HCL: Hairy cell leukemia, ATL: Adult T cell leukemia, CTCL: Cutaneous T cell lymphoma, T-ALL: T cell acute lymphocytic leukemia, NK: Natural killer, HL: Hodgkin's lymphoma, ALCL: Anaplastic large-cell lymphoma, AML: Acute myeloid leukemia, Her2: Human Epidermal Growth Factor Receptor 2

Combotox was first demonstrated to be effective in a Daudi-Lymphoma (CD19+/CD22+) SCID mouse model, where the use of both antibodies showed a synergistic killing effect, promoting survival to the equivalent killing of ≥5 logs of tumor cells, more than 1 log greater than RFB4-dgRTA alone, and at least 3 logs greater than HD37-dgRTA alone.[18] These results led to a 22 patient Phase I clinical trial for patients with advanced or refractory Non-Hodgkin's B-Cell Lymphoma (NHL). The results were modestly encouraging, with 9% of patients achieving a partial response (PR) and 23% a minor response (MR). The main side effects from the drug were complications caused by a Vascular Leak Syndrome (VLS). The symptoms of VLS include edema, pulmonary congestion, dyspnea, weight gain, anemia, hypoalbuminemia, hypotension, and in extreme cases multi-organ failure and death. Severe hemolytic uremic syndrome (HUS) was also observed. While three patients died during the trial, only two of these deaths could be directly attributed to Combotox. Additionally, 30-35% of patients developed human anti-mouse and/or anti-RTA antibodies (HAMA and/or HARA). Interestingly, toxic side effects were inversely correlated with the presence of circulating tumor cells (CTCs). Patients with ≥50 CTCs/mm^3^ tolerated maximum dosing without major toxicities, while those with <50 CTCs/mm^3^ experienced more serious adverse events. Patients with a history of autologous or allogeneic bone marrow transplant (BMT), or radiation therapy also had a higher mortality.[19]

Since relapsed or refractory precursor-B acute lymphoblastic leukemia (pre-B ALL) usually has circulating blasts cells, Combotox was next tested in this malignancy. Combotox demonstrated its efficacy in *in vitro* experiments with pre-B ALL lymphoblasts taken from pediatric patients,[20] as well as *in vivo* early and late disease pre-B ALL murine models.[21] In a Phase I pediatric clinical trial for refractory pre-B ALL (n=17), Combotox treatment resulted in a complete remission (CR) for 18% of patients and hematological improvement (HI) in 35% of patients. The main dose-limiting toxicity (DLT) was Graft Versus Host Disease (GVHD) in two patients with a history of prior stem cell transplantation (SCT). The most common adverse events were mouth sores, rashes, and hyperbilirubinemia. A case of pancreatitis and anaphylaxis each was also reported. Two patients died during the trial, and both deaths were attributed to a high leukemic burden. The maximum tolerated dose (MTD) was determined as 5 mg/m^2^ per dose for up to three doses. The rate of HAMA/HARA immunogenicity was 18%, about half of that from the NHL adult trial.[22]

These encouraging results informed a subsequent Phase I trial of single agent Combotox in adults with B-lineage ALL. In this trial (n=17), treatment with Combotox led to specific reductions in peripheral leukemic blasts in most patients: even though only a 13% (n=2) PR rate was observed, 5 patients (31%) experienced a hematological response resulting in an overall response rate (ORR) of 31%. The MTD of Combotox was three doses of 7mg/m^2^ every other day. The DLT in this trial was VLS. Other serious side effects at grade 3 or higher included elevated liver function tests. HAMA/HARA immunogenicity was 6%, the lowest heretofore reported rate in any Combotox clinical trial.[23] These trials demonstrated that Combotox has specific activity against B-lineage ALL blasts, but single agent treatment may not be sufficient in this rapidly progressive disease. Subsequent *in vitro* studies were conducted to test its efficacy in combination with chemotherapy in a NOD mouse model of advanced ALL. This murine xenograft experiment revealed that sequential administration of Combotox with cytarabine (Ara-C) is superior to concurrent administration and improved survival over single agent therapy. These results informed the design of a Phase I clinical trial for adults with relapsed or refractory ALL that is presently accruing patients (clinicaltrials.gov NCT01408160).[24]

### Brentuximab Vedotin is an immunotoxin against CD30 that is approved for use in Hodgkin's lymphoma and systemic anaplastic large cell lymphoma

SGN-35 (cAC10-vcMMAE; Seattle Genetics) is the most recent ADC to be approved by the FDA.[25] Going by either the generic name Brentuximab Vedotin, or the trade name, Adcetris™, SGN-35 consists of the chimeric monoclonal IgG1 cAC10 (SGN-30) antibody, which is specific for human CD30, conjugated to a toxin, monomethyl auristatin E (MMAE).[26] CD30, part of the tumor necrosis factor (TNF) receptor family, is an ideal target for antibody-based therapy, as it is highly expressed on HL and sALCL, while restricted to the immune system, specifically to activated lymphocytes (Table 2). MMAE is a synthetic analog of dolastatin 10, a highly potent natural antimitotic agent that inhibits tubulin polymerization. The third component is the stable cathepsin B cleavable valine-citrulline dipeptide linker, with a *p*-aminobenzylcarbamate spacer put in between the linker and toxin. *In vitro* cytotoxicity assays on the CD30+ Karpas-299 ALCL and L540cy HL cell lines showed that SGN-35 was both highly potent and antigen-specific. It induced G2/M phase growth arrest quickly followed by apoptosis. Additionally, the drug was shown to be highly stable in human plasma (due to the unique linker), as less than 2% of the drug was released after a 10-day incubation. *In vivo* experiments in xenograft SCID mouse models of sALCL and HL, and a disseminated sALCL SCID mouse model, all demonstrated dose dependent tumor regression and a high tolerance for the drug.[27] Further *in vitro* studies showed that the most effective form of SGN-35 would optimally include four molecules of MMAE conjugated to each antibody.[28] The linker in SGN-35 was examined *in vivo* and was determined to be far more stable than previously used disulfide and hydrazone linkers, with a half-life of almost ten days.[29] The method of MMAE entry into the cell was elucidated via *in vitro* studies, which showed that both the mAb and MMAE were internalized into the cell via clathrin-dependent mechanisms, while the drug was released via cathepsin B and other lysosomal cysteine proteases.[30] Finally, in an *in vivo* L540cy HL SCID mouse model, SGN-35 was combined with either ABVD (doxorubicin, bleomycin, vinblastine, and dacarbazine) or gemcitabine as a combination therapy, and showed synergistic anti-tumor activity.[31]

These results encouraged clinical trials testing the efficacy of SGN-35 in different CD30-positive malignancies. In a Phase I clinical trial with 45 patients with either refractory HL (n=42), sALCL (n=2), or angioimmunoblastic T-cell Lymphoma (n=1), 17 patients had a objective response (OR=CR + PR; 38%), with 11 patients having a CR (24%), while 19 patients (42%) had stable disease (SD). Among patients treated at the MTD (1.8mg/kg), the OR rate was 50%. Additionally, tumor regression was noted in 86% of evaluable patients (36/42), while 81% of patients with disease symptoms at baseline (13/16) experienced symptom resolution. Serious side effects ≥grade 3 included one case each of thrombocytopenia, prostatitis, febrile neutropenia, hyperglycemia, acute renal failure, and presumed sepsis leading to death. The more common grade 1/2 side effects included fatigue, pyrexia, diarrhea, and neutropenia. Peripheral sensory neuropathy (PSN) and associated adverse events were observed in 16 patients (36%).[32]

Since then, two Phase II clinical trials have been reported. One hundred and two patients with refractory HL post autologous SCT were given 1.8 mg/kg SGN-35 every 3 weeks. The ORR was 75% and the CR rate was 34%, while tumor reduction was seen in 94% of patients. Thirty-one of 102 patients were alive and free of documented progressive disease after a median observation time of 18.5 months. Serious side effects ≥grade 3 included PSN, neutropenia, thrombocytopenia, and anemia. Other common adverse events included nausea, fatigue, diarrhea, pyrexia, vomiting, arthralgia, pruritus, myalgia, and peripheral motor neuropathy (PMN).[33] Similarly, 58 patients with refractory sALCL were given 1.8 mg/kg SGN-35 every 3 weeks. The ORR was 86%, with a 57% CR rate while tumor reductions were seen in 97% of patients. In the 17 patients with B-symptoms, 82% achieved resolution in their symptoms. Serious side effects ≥grade 3 were similarly PSN, neutropenia, thrombocytopenia, anemia, fatigue, and pain in the extremities. Interestingly, 4 patients had tumor flares.[34] In an attempt to reduce toxicity and increase potency, 44 patients diagnosed with refractory HL (n=38), sALCL (n=5), or peripheral T-cell lymphoma not otherwise specified (PTCL-NOS) were enrolled in a weekly dosing Phase I clinical trial. SGN-35 elicited a response in 24 patients (59%), CR in 14 patients (34%), and tumor reduction in 93% of evaluable patients (41/44). Of the 7 patients with B-symptoms at baseline, 6 had symptom resolution. Again, serious side effects ≥grade 3 included PSN, anemia, and neutropenia, but also PMN, hyperglycemia, diarrhea, vomiting, hypokalemia, and -magnesemia. Overall, this study showed that SGN-35 might be more effective at a weekly dosing, but also had increased adverse events, especially related to peripheral neuropathy.[35]

Given its impressive activity, the use of SGN-35 is currently being explored in several clinical trials in the upfront setting combined with cytotoxic agents, as single agent and in combination with other drugs for relapsed refractory CD30+ malignancies, as well as maintenance therapy post-induction chemotherapy or following SCT. Preliminary results have been very promising, especially in the upfront treatment of classical HL when combined with doxorubicin, vinblastine and dacarbazine (“A+AVD”), where CR rates approached 95% in a Phase II trial (Ansell SM et al. ASH 2012), and for frontline treatment for patients with CD30+ mature T- and NK-cell lymphomas as “A+CHP” (SGN-35 plus cyclophosphamide, doxorubicin, prednisone) where an ORR of 100% and a CR rate of 88% was observed (Fanale MA et al. ASH 2012). Both combinations are currently explored in ongoing phase III clinical trials (clinicaltrials.gov NCT01712490 and NCT01777152).

Although a great addition to lymphoma therapy, a potential caveat for the future widespread use of SGN-35 might be the increased risk for pulmonary toxicity, especially when used with other agents such as bleomycin (Ansell SM et al. ASH 2012) or gemcitabine, as well as the development of progressive multifocal leukoencephalopathy (PML), for which the FDA has issued a black box warning.[36] An interesting observation is that retreatment with SGN-35 might result in responses even in patients who have previously progressed on therapy with brentuximub vedotin (Bartlett N et al. ASCO 2010). This observation, though, will need confirmation in a larger more systematically conducted clinical trial.

### Trastuzumab emtansine targets her-2-neu receptors in breast cancer

Trastuzumab emtansine (T-DM1; Kadcycla™) is the most recently FDA approved ADC and indicated for the treatment of Human Epidermal Growth Factor Receptor 2 (HER2) positive metastatic breast cancer. Based off of the HER2 (EGFR2, neu) targeting mAb trastuzumab (Herceptin™), T-DM1 consists of the antibody trastuzumab conjugated to the anti-mitotic maytasanoid N(2')-deacetyl-N(2')-(3-mercapto-1-oxopropyl)-maytansine (DM1) via an *N*-[maleimidomethyl] cyclohexane-1 carboxylate (MCC) linker. DM1 is a powerful microtubule-depolymerizing agent with action thought to be similar to that of the vinca alkaloid class of chemotherapeutics. The linker is connected to DM1 via a nonreducible thioether bond, while it connects to the mAb at ε-lysine side chains. This novel linker was originally seen to be superior to a number of disulfide bond based linkers, as initial reports showed that the MCC linked mAbs had a lower clearance rate, longer half-life, and stronger anti-tumor effects along with reduced toxicity in several *in vivo* (but not *in vitro*) models of Her2 positive breast cancer.[37] However, recent research seems to contradict this observation, as comparisons between MCC and *N*-succinimydyl 4-(2-pyridyldithio)-pentanoate (SPP)-reducible disulfide based linkers have shown the opposite: *in vitro*, MCC-T-DM1 had a stronger cytotoxic effect compared to SPP-T-DM1, but this did not translate to more potent tumor reduction in *in vivo* models of breast cancer.[38] To further complicate this scenario, new THIOMAB technologies have been developed that allow for a more homogenous population of ADCs. THIOMABs are antibodies that can be used as intermediates to produce ADCs secondary to an engineered unpaired cysteine residue on each heavy chain.[39]

Most ADCs are in fact a heterogeneous mixture of mAbs, with different numbers of toxic drugs attached to each mAb.[40] For example, MCC-T-DM1 exists in a heterogeneous form, ranging from 0-7 DM1 molecules on each mAb, with a mean of 3.3 per mAb. The thio-T-DM1 is constructed with a nonreducible bis-maleimido trioxyethylene glycol (BMPEO) linker that attaches to the engineered cysteines on the thio-mAb. This form of T-DM1 has an average of 1.8 DM1 drugs per mAb, with 90% of the population having 2 DM1 molecules per mAb. *In vitro* studies proved the equivalent efficacy of this newer ADC, while *in vivo* models showed that it was more effective at reducing tumor burdens while having a safer profile in rats and cynomolgous monkeys.[41]

Much preclinical research has been conducted on T-DM1. It has proven to be effective *in* and *ex vivo* at killing trastuzumab-resistant breast, and other epithelial tumor cell lines. The dominant mechanism of cytotoxicity appears to be through apoptosis and cell lysis, and not cytostatic G2-M phase arrest as would be expected from its drug class. Protease inhibitors blocked its activity, implicating that T-DM1 is broken down in a lysosomal dependent manner to an active catabolite, lysine-MCC-DM1, which has anti-microtubule activity, but cannot penetrate the cell membrane, thereby lessening the bystander effect.[37] *In vitro* studies have also shown that DM1 itself is far more potent than commonly used cytotoxic agents in breast cancer such as paclitaxel or doxorubicin, and that T-DM1 retains the same mechanisms of action as trastuzumab alone: they both have equivalent Her2 binding affinities, antibody-dependent cell-meditated cytotoxicity (ADCC) activation, Akt inactivation, and HER2 ectodomain shedding induction, implying that T-DM1 may be able to replace Herceptin™ as a frontline chemotherapy. T-DM1 was also effective against a variety of trastuzumab and lapatinib resistant, and PI3K-pathway activated, cell lines *ex* and *in vivo*.[42] Recently, it has been discovered that T-DM1 can also cause cytotoxicity through mitotic catastrophe.[43]

Numerous clinical trials exploring the use of T-DM1 in breast cancer as a single agent or in combination with cytotoxic and Her2-directed therapy have been completed by now. In a Phase I clinical trial in advanced HER2+ breast cancer patients (n=24), increasing doses (0.3-4.8 mg/kg) of T-DM1 were administered once every 3 weeks. The OR rate was 25% without any CRs, while in the 15 patients who received the drug at the MTD of 3.6 mg/kg, 73% had clinical benefit (OR and stable disease) at 6 months. Serious side effects ≥ grade 3 included thrombocytopenia and pulmonary hypertension. The most common adverse events were thrombocytopenia, elevated hepatic transaminases, fatigue, anemia, and nausea. Fortunately, only 1of 22 patients evaluated developed anti-therapeutic antibodies (ATA). The MCC linker was shown to be quite stable as well: patients exhibited a 70-fold difference in T-DM1 versus DM1 plasma concentrations.[44]

Based on this data, a Phase II trial was initiated treating 112 patients with HER2 positive metastatic breast cancer (MBC) who had received prior Her2 based therapy, with T-DM1. The ORR was 26%, with no CRs, and a median progression-free survival (PFS) of 4.6 months. Only 74 of the 95 enrolled patients had their Her2 status retroactively confirmed via FISH (fluorescent in-situ hybridization) or IHC (immunohistochemistry). In these 74 patients the ORR was higher (34%) and the median PFS was 8.2 months. Serious side effects ≥grade 3 included hypokalemia, thrombocytopenia, and fatigue. The most common adverse events (AE) were fatigue, nausea, headache, pyrexia, epistaxis, constipation, cough, diarrhea, vomiting, hypokalemia, arthralgia, extremity pain, anemia, and dyspnea. Interestingly, 31% of patients developed ophthalmological AEs. Seven out of 108 evaluable patients tested positive for ATA.[45]

Another recently completed Phase II trial of single agent T-DM1 enrolled 110 heavily pretreated patients. The ORR was 35%, with no CRs, and a median PFS of 6.9 months. Again, by FISH testing Her2 positive status was confirmed for only 80 out of 95 patients. For this subgroup, the ORR was 41% with a median PFS of 7.3 months. Serious side effects ≥grade 3 included thrombocytopenia, fatigue, and cellulitis, with 8% of patients reporting serious hepatic toxicity. Again, the most common AEs were fatigue, thrombocytopenia, nausea, elevated AST, constipation, pyrexia, epistaxis, headache, hypokalemia, decreased appetite, dry mouth, and anemia. Six out of 108 patients developed ATA.[46] In an effort to increase drug exposure, while preserving a similar side effect profile, a weekly dosing schedule of T-DM1 was explored in a Phase I trial (n=28). The MTD was determined to be 2.4 mg/kg, with an ORR of 46% and no CRs. AEs were very similar to the other trials, while no patients developed ATAs.[47]

In a retrospective analysis of the above mentioned 3 trials,[36-38] the effect of prior T-DM1 treatment on subsequent treatment outcomes was explored. Clinical outcomes of 15 patients who had received T-DM1 and were now receiving other therapies were examined to determine if T-DM1 could adversely affect follow-up treatment, especially when giving Herceptin™ or Lapatinib. There did not appear to be any negative consequences in receiving T-DM1, but the interpretation is limited by the small sample size.[48] Additionally, to better understand the mechanism behind T-DM1 induced thrombocytopenia, the main DLT of the drug, a novel high-content, quantitative, live-cell imaging technique was used. The investigators demonstrated that T-DM1 is taken up by megakaryocytes (MKs) via a non-EGFR-dependant pathway (MKs are HER2-). The microtubule targeting of DM1 in MK inhibits pro-platelet production and MK differentiation, induces abnormal tubulin organization, and suppresses microtubule dynamic instability.[49]

The results of the landmark phase III EMILIA trial lead to the FDA approval of the drug for Her2-positive MBC patients previously treated with a trastuzumab and a taxane. In this trial, T-DM1 was compared to combination therapy with lapatinib, an oral Her2 inhibitor, and capecitabine in 991 patients with advanced HER2 positive breast cancer who had previously been treated with trastuzumab and a taxane. Treatment with T-DM1 resulted in significantly improved ORR (43.6% versus 30.8%), prolonged progression-free (median PFS 9.6 months versus 6.4 months; Hazard Ratio [HR] 0.65; p<0.001), and overall survival (median OS 30.9 months versus 25.1 months; HR= 0.68; p<0.001). Additionally, compared to lapatinib plus capecitabine, patients experienced less grade 3 toxicities (41% versus 57%).[50] In the meantime many clinical trials exploring the use of this promising agent in the upfront metastatic and adjuvant setting alone and in combination with other agents are currently undergoing. A recently reported randomized Phase II clinical trial compared T-DM1 to a combination of trastuzumab and docetaxel (HT) as first-line therapy for patients with Her2-positive MBC and appeared better tolerated and more effective.[51] Given these results T-DM1 seems bound to become the preferred treatment for Her2+ MBC.

### Moxetumomab Pasudotox targets CD22-positive lymphoid malignancies

Moxetumomab Pasudotox (HA22, CAT-8015) is a second-generation recombinant Immunotoxin (RIT) which targets CD22 and is derived of the same RFB4 monoclonal antibody used for Combotox. HA22 is a high-CD22 affinity version of BL22 (CAT-3888), another RIT. BL22 itself was based off of an ADC that combined the antibody RFB4 linked to a Pseudomonas Exotoxin (PE). PE exerts its cytotoxic effect on cells by ADP-ribosylating Elongation Factor 2 (EF2), a protein integral for translation, thereby inhibiting protein synthesis and leading to cell death. To reduce non-specific toxicity, the PE toxin was modified by removing its cell-binding domain, leading to the development of PE38.[52] To improve its pharmacokinetic profile, increase its potency, and facilitate production, the IT was turned into an RIT. The variable domain (Fv) portions of the mAb were cloned, and a disulfide linkage was added between the heavy (V_H_) and light (V_L_) chain to increase stability. Then the V_H_ domain was genetically fused to the PE38 toxin via the C3 (six amino-acid) connector. This process eliminated the need for a linker. BL22 is highly cytotoxic to CD22+ cell lines and prevents tumor growth *in vivo* in a Burkitt Lymphoma mouse model.[53] Preclinical mouse and primate models showed strong CD22-specific and dose-dependent anti-tumor activity for an alternate day treatment cycle for three doses, while also being well tolerated.[54] Further *in vitro* studies with cells from 28 cancer patient samples showed that BL22 was cytotoxic in around half of the patients' samples. Cytotoxicity and CD22 expression were positively correlated.[55]

The initial results from the first Phase I clinical trial of BL22 in patients with Hairy Cell Leukemia (HCL) were very promising. Sixteen out of the 31 patients on the trial had refractory HCL, and in this cohort of patients, 69% had CRs with an ORR of 81%. Of the 11 CRs, only one had signs of Minimal Residual Disease (MRD) in the bone marrow. Unfortunately some serious side effects associated with BL22 were observed, including Cytokine Release Syndrome (CRS) and Hemolytic Uremic Syndrome (HUS). To prevent CRS, patients were subsequently pre-treated with daily Rofecoxib and Infliximab a week before and after treatment. This strategy appeared to prevent further cases of CRS. Less serious side effects included hypoalbuminemia, elevated aminotransferase levels, nausea, myalgia, edema, and elevated creatinine levels, which were all reversible. Twenty-five percent of the patients developed neutralizing antibodies to the IT.[56] In the meanwhile, HA22 was developed from BL22 as an RIT with a greater than 10 times affinity for CD22. This higher affinity also resulted in greater cytotoxicity toward CD22+ cell lines and enabled researchers to examine its efficacy in hematological malignancies with lower CD22 expression.[57] Further versions of CD22 with even greater affinity and cytotoxicity were developed, although they so far have not been clinically pursued.[58, 59] Additional variants of HA22 have been tested that have increased stability with reduced immunogenicity and antigenicity, including HA22-8X,[60] HA22-LR,[61] HA22-LR-8M,[62] and HA22-LR-L010.[63] Additionally, the mechanisms of BL22 cytotoxicity were further elucidated, with experiments showing that BL22 can induce apoptosis via a caspase-3-like protease, in addition to its ability to inhibit translation.[64] Further research has shown that BL22 leads to PARP (Poly (ADP-ribose) polymerase) cleavage and both caspase-3 and caspase-9 activation in patient CLL (chronic lymphocytic leukemia) samples.[65] The sensitivity of cells to apoptosis, which is inversely proportional to Bcl2 expression, was seen as a key determinant in the efficacy of BL22 on Mantle Cell Lymphoma (MCL) cell lines, instead of being correlated with CD22 expression. This suggests Bcl2 overexpression as a possible method for cells to develop resistance against BL22.[66]

The final results from the first BL22 Phase I clinical trial in B-Cell malignancies were as encouraging as the initial results for HCL patients. A total of 46 patients with various B-cell malignancies (31 with HCL, 11 with CLL, and 4 with other NHL) were enrolled. HCL patients had a high CR rate of 61% and ORR of 80%, however, the response was much more muted in the other patients, with only 3 out of 11 CLL patients having a marginal response. Although originally, prophylactic anti-inflammatory drugs were used to prevent CRS, it soon became apparent that HUS proved to be a more serious safety issue. In order to prevent HUS, prophylactic intravenous fluid was administered to diminish renal toxicity. This measure, along with reducing the dose of BL22, appeared to ameliorate the issue. Otherwise, the safety profile was similar to that of the initial Phase 1 study in HCL, with 35% of HCL, but no CLL or NHL patients developing neutralizing antibodies.[67] In a Phase II trial testing BL22 in 36 patients with refractory HCL, CRs were seen in 47% of patients (with 18% having MRD), and an ORR of 72%. Interestingly, smaller spleen size was highly correlated with an improved response. Serious associated toxicities included transaminitis, hypoalbuminemia, fever, thrombocytopenia, VLS, proteinuria, anemia, and hypoxia. All of these toxicities were reversible and did not constitute a DLT or were associated with HUS. The most common AEs were hypoalbuminemia, transaminitis, edema, myalgia, proteinuria, fatigue, nausea, and fever. Neutralizing antibodies were seen in 11% of patients.[68]

BL22 was less successful, however, in treating pediatric pre-B ALL. In a Phase I clinical trial for pediatric patients with refractory B-cell malignancies (n=23), 21 of whom had pre-B ALL, there were no CRs or PRs, although there was transient clinical activity seen in 70% of patients. Serious side effects at ≥grade 3 included transaminitis and myelosuppression. The most common toxicities were hypoalbuminemia, transaminitis, and proteinuria. No cases of HUS or VLS were observed, and no DLT was seen. Although neutralizing antibodies were found in 13% of patients, the drug appeared to be much better tolerated in children than adults.[69] To increase efficacy, the use of Bryostatin I, a weak chemotherapeutic agent that upregulates CD22 expression, was explored. In *in vitro* CLL and MCL patient samples,[70] Bryostatin I was administered before BL22 and resulted in increased efficacy. Sequential administration of Bryostatin I followed by BL22 may be a useful method to improve results for the treatment of hematological malignancies that have lower levels of CD22 expression. Another way to increase efficacy of CD22-directed therapies may be by using HA22, which has a much higher affinity for CD22. HA22 has been tested in an *in vivo* mouse model of primary intraocular lymphoma (PIOL), where a single dose injection of HA22 into the eye was enough to cause complete tumor regression, with minimal associated eye toxicity.[71] In pre-clinical studies, HA22 was significantly more cytotoxic than BL22 toward CD22+ cell lines, and more effective in reducing tumor volumes in a Burkitt Lymphoma mouse model.[72] This improved efficacy for HA22 compared with BL22 was also demonstrated in cell cultures of pediatric pre-B ALL patient samples.[73] A potential mechanism for HA22 resistance in ALL patients has been described. *In vitro* studies on resistant ALL cell lines revealed that a CpG island in the promoter region of DPH4, which codes for the diphthamide biosynthesis protein and is crucial for PE toxin mediation, is hypermethylated, leading to downregulated levels of the protein. This DPH4 downregulation is transient and dependent on the presence of HA22, and can be overcome by pretreatment with the methylation inhibitor azacytidine.[74] More recently, a second mechanism for resistance has been discovered in a resistant Burkitt NHL cell line, where a mutation has led to the deletion of WDR85, another gene that is necessary for dipthamide synthesis (Wei H et al. ASH 2012).

In a Phase I clinical trial with 28 refractory HCL patients, every other day HA22 treatment for 3 doses led to CRs in 46% of patients, with an ORR of 86%. Out of 9 patients who achieved a CR, only one was positive for MRD. Like before, clinical success was inversely proportional to spleen size, with patients having a prior splenectomy suffering worse outcomes. Serious side effects ≥grade 3 included lymphopenia, γ-glutamyltransferase (GGT) elevation, and leukopenia, although none were considered serious enough to be considered a DLT. The most common AEs were hypoalbuminemia, transaminitis, limb/head and neck edema, headache, hypotension, and nausea. Neutralizing antibodies to the toxin were reported in 38% of patients.[75] A recent update to this study involving 20 more patients (n=42) confirms the above findings, with 55% achieving a CR, and an ORR of 88%. Of the 21 CRs evaluable for MRD, only 4 were positive (Kreitman RJ et al. ASCO 2012). A phase I trial treating pediatric ALL with HA22 at 6 doses every other day per 3-weekly cycle is still ongoing, but preliminary results appear promising. ORs were reported for 5 of 17 evaluable patients (29%), with 4 (24%) achieving a CR. Hematological activity (HA), as defined by a ≥50% reduction in blasts and/or improvement in neutrophil and/or platelet counts, was seen in 7 patients (41%). Serious VLS was reported in two of the first seven patients, although this toxicity has been ameliorated when dexamethasone was added as pre-treatment. Anti-moxetumomab pasudotox neutralizing antibodies were reported in 14% of patients (Wayne AS et al. ASH 2011). HA22 is currently being evaluated in at least 2 ongoing phase I clinical trials (ClinicalTrials.gov Identifier: NCT00586924 and NCT00659425).

### Inotuzumab Ozogamicin is showing promise in treating CD22+ ALL and NHL

Inotuzumab Ozogamicin (INO; CMC-544) is another anti-CD22 directed ADC. Its antibody is based off of the humanized IgG4 G5/44 mAb (not RFB4). CMC-544 is a close relative of Gemtuzumab Ozogamicin (GO; Mylotarg™), an anti-CD33 mAb with the same linker and toxin as CMC-544. GO was the first ADC approved by the FDA for the treatment of AML,[76] but was later voluntarily withdrawn from the US market because of concern about its toxicity (mainly sinusoidal obstruction syndrome).[77] Of note, recent findings support GO's usefulness and reduced toxicity when given in lower doses during induction therapy, by increasing disease-free survival specifically in older patients.[78, 79] The IgG4 G5/44 mAb was chosen, as it had the best combination of high CD22 affinity combined with a high rate of internalization. The mAb is joined to its toxin via the acid hydrolysable 4-(4'-acteylphenoxy)butanoic acid (AcBut) linker. This linker was proven to be more effective than a more stable amide linker in both *in vitro* cytotoxicity and *in vivo* anti-tumor assays.[80] The toxin is *N*-acetyl-γ-calicheamicin dimethyl hydrazide (CalichDMH), which is derived from the γ-calicheamicin antitumor antibiotic naturally produced by the bacterium micromonospora echinospora. This extremely potent product mediates its cytotoxicity by binding DNA in its minor groove, then undergoing thiol-dependent structural changes in its enediyne moiety to generate a di-radical, which abstracts hydrogens from the phosphodiester backbone of DNA, leading to double stranded breaks in the DNA, and ultimately cell death. There is an average of 5-7 moles of toxin per mole of mAb. CMC-544 is seen as more of a targeted chemotherapy as opposed to an immunotherapy, due to the weakness of its mAb, which has nearly no efficacy on its own, owing to its inability to fix complement or initiate ADCC. However, CMC-544 was even more cytotoxic against CD22+ B-lymphoma cell lines than unconjugated CalichDMH, and had strong dose-dependent anti-tumor activity against small and large B-cell lymphoma (BCL) xenografts.[81]

Further studies showed that CMC-544 was also active in both early and late stage disseminated BCL murine models, while Rituximab, a chimeric anti-CD20 mAb, was only effective, and to a lesser degree, at the early stage.[82] Preclinical research showed that CMC-544 could have an additive or synergistic effect when combined with Rituximab. This could be due to their different modes of action, as Rituximab relies on ADCC/fixing complement for its anti-tumor activity, which CMC-544 is unable to perform. Importantly, CMC-544 caused significant tumor regression in a Rituximab-refractory established tumor model.[83] CMC-544 was also found to have strong cytotoxicity against ALL cell lines, along with potent dose dependent anti-tumor activity in both subcutaneous xenograft and disseminated ALL murine models.[84] Finally, CMC-544 demonstrated greater efficacy than either of the frontline combination NHL therapies, CHOP (cyclophosphamide, doxorubicin, vincristine, and prednisone) or CVP (CHOP without doxorubicin) in both *in vitro* and *in vivo* assays. Importantly, CMC-544 retained activity in CHOP/CVP-refractory tumor models, while also showing increased potency when given in combination with CVP (but not CHOP, which was too toxic).[85]

Further *in vitro* studies showed that CMC-544 efficacy was inversely correlated with P-glycoprotein (P-gp) expression, an efflux pump responsible for multi-drug resistance (MDR) in CLL and NHL cell lines, as well as in patient samples. However, this could be overcome by combining CMC-544 with the MDR modifiers PSC833 or MS209.[86] Additionally, it was discovered that CD22 and CD55 expression decreased after CMC-544 treatment in both BCL cell lines and patient samples, while CD20 and CD59 levels remained the same. This correlated with an increased efficacy in the complement dependent cytotoxicity (CDC) of Rituximab that was seen only when given after CMC-544 treatment, and not when given simultaneously.[87] Research with pre-B ALL patient samples showed that the large variation in sensitivity to CMC-544 was directly correlated with sensitivity of the cells to free calicheamicin and how quickly CMC-544 could be internalized into the cells, but was not dependent on extracellular CD22 levels or the ability of the cells to renew their CD22 expression.[88]

The first Phase I trial of CMC-544 involved 79 patients with refractory NHL, with the majority (35 each) having either follicular lymphoma (FL) or diffuse large B-cell lymphoma (DLBCL). The MTD was established at 1.8 mg/m^2^ given once every 3-4 weeks, with the DLT being thrombocytopenia. While the ORR was only 39%, the ORR at MTD for FL was 68%, with 32% CRs and a median PFS of 317 days, while for DLBCL it was a lower 15%, with 7.7% CRs and a median PFS of 49 days. Serious side effects at ≥grade 3 included thrombocytopenia, neutropenia, leukopenia, asthenia, arthralgia, and fever. The most common AEs were thrombocytopenia, asthenia, nausea, neutropenia, fever, elevated AST levels, abdominal pain, and anorexia. While thrombocytopenia was a major issue, without a known obvious mechanism for its occurrence, there were no major hemorrhages reported.[89] Another smaller Phase I trial, involving 13 Japanese patients with refractory FL who had previously been treated with Rituximab, had similar findings. The ORR was 85%, with 54% CRs. Serious side effects ≥grade 3 included thrombocytopenia, lymphopenia, neutropenia, leukopenia, hyperbilirubinemia, and hypokalemia. The most common AEs were thrombocytopenia, leukopenia, neutropenia, elevated AST levels, anorexia, and nausea.[90]

A more recent Phase I trial, also done in Japan, which combined CMC-544 treatment one day following Rituximab every 4 weeks in 10 refractory NHL patients (6 of whom had FL) also showed promising results. The ORR was 80%, with 70% CRs, while the CR rate in FL patients was 83%. Serious side effects ≥grade 3 included thrombocytopenia, neutropenia, lymphopenia, leucopenia, hypophosphatemia, and elevated AST levels. The most common AEs were thrombocytopenia, elevated transaminase levels, leukopenia, nausea, neutropenia, and lymphopenia. Importantly, Rituximab did not induce any major changes in the pharmacokinetic or safety profile of CMC-544 than when CMC-544 was used as a monotherapy.[91] Most recently, Fayad and colleagues reported a combined dose-escalation Phase I/II trial of R-INO (rituximab and INO) in patients with relapsed FL and DLBCL, and refractory aggressive NHL (n=118). At the MTD (Rituximab 375mg/m^2^ on day 1, INO 1.8mg/m^2^ on day 2, given every 4 weeks for up to 8 cycles), the ORR was 87%, 74%, and 20% respectively for FL (n=39), DLBCL (n=42) and refractory NHL (n=30), with a 2-year progression-free survival (PFS) of 68%, and 42% for FL and DLBCL, respectfully. Thirty percent of patients treated experienced serious AEs, which included pneumonia, sepsis, thrombocytopenia, nausea and/or vomiting, peripheral edema, chest pain, dizziness, infection and nodular regenerative hyperplasia. Treatment at MTD was discontinued in 43% secondary to toxicities, most commonly secondary to thrombocytopenia or hyperbilirubinemia.[92]

The addition of INO to chemotherapy has also been explored in another Japanese Phase 1 study (Ogura M et al. ASH 2011), with the expansion cohort at the MTD having been recently updated. Ogura and colleagues identified the MTD as full dose R-CVP combined with INO at 0.8mg/m^2^ given on day 2 of a 3-weekly cycle. In the expansion cohort, 32 patients with relapsed or refractory B-cell NHL [FL (n = 15), MCL (n = 1), and DLBCL (n = 16)] were treated at MTD. The ORR was 100% (53% CR) and 605 (7% CR) for patients with FL and DLBCL respectively. The most commonly described AE of any grade were thrombocytopenia, leukopenia, neutropenia, fatigue, constipation, and nausea; the most common grade ≥3 AEs included cytopenias and transaminitis with 1 death secondary to pneumonia in a neutropenic patient (Ogura M et al. ASH 2012).

Another trial looked at Rituximab plus INO (R-INO) salvage therapy followed by autologous SCT (ASCT) for 61 patients with relapsed/refractory DLBCL. Common adverse events during R-INO treatment were again cytopenias, transaminitis, fatigue, pyrexia, and vomiting (24%). Two patients had veno-occlusive disease of the liver (VOD) after ASCT. Fifty-four evaluable patients received a median of 3 cycles of R-INO. The ORR after R-INO was 35% (24% CR, 11% PR), 13% had SD, and 50% progressive lymphoma. Prior response to the most recent therapy was predictive of response to R-INO. Stem cell mobilization and stem cell collection after R-INO appeared acceptable. Six- and 12-month PFS rates for all treated patients were 31% and 13%, respectively (median PFS 2.6 months), and the median OS was 10 months. For patients who underwent ASCT, the 6- and 12-month PFS rates were 79% and 35%, respectively (median PFS 10 months) and the median OS was not reached (Wagner-Johnson N et al. ASH 2011). Multiple other trials with INO in NHL, in combination with Rituximab or chemotherapy, in the upfront and relapsed setting, are planned or ongoing.

In addition to the treatment of NHL, INO shows promise also for ALL. A Phase II trial involving 49 adult and pediatric patients with refractory ALL showed encouraging results. Eighteen percent of patients achieved a CR, and 39% had a marrow CR (<5% lymphoblasts), resulting in an ORR of 57%. The median OS was 5.1 months for all patients, and 7.9 months for responders. Serious side effects ≥grade 3 included thrombocytopenia, neutropenia, fever, and hyperbilirubinemia, which was reversible in most cases. The most common adverse events were thrombocytopenia, neutropenia, elevated ALT/AST levels, fever, hypotension, and hyperbilirubinemia.[93] Two trials explored a weekly schedule of INO for patients with relapsed refractory B-cell ALL (DeAngelo D et al. ASH 2012; Jabbour E et al. ASCO 2012). Responses were consistent in both trials and observed across all INO doses. The ORR ranged around 82% with a CR and CRi (incomplete CR) rate ranging between 45-50%. At the same time the toxicities appeared similar in nature (hematological, liver and gastrointestinal), but possibly less severe and/or frequent.

Given these encouraging early results in both B-cell NHL and ALL, INO is currently being tested in multiple Phase I, II, and III trials alone, in combination with Rituximab, and with other chemotherapeutic regimens (e.g. GemOx [gemcitabine and oxaliplatin], GDP [gemcitabine, dexamethasone, and cisplatin], temsirolimus, and CVP).

## CONCLUSIONS

The five types of ADCs described here in detail illustrate some of the many different strategies research groups are taking to bring ADCs from bench to bedside (Table 2). The two most recently FDA approved ADCs, Brentuximab vedotin and Trastuzumab emtansine, were welcome by clinicians with great anticipation. These ADCs were developed using novel peptide and thioether linkers, respectively, giving them an advantage over older ADCs using linkers with less stability, and therefore causing more collateral damage secondary to systemic off-target effects by free toxin. Following the biotechnological progress in ADC development there has to be a similar progress in finding the best dosing strategies and combination with other drugs to maximize their efficacy. The inherent specificity of ADCs may serve as the perfect complement to the broad based killing effects of standard cytotoxic chemotherapies, as ADCs are able to destroy slower growing and even quiescent cancer cells that are more resistant to chemotherapy and most likely responsible for relapse and refractoriness. Determining the optimal dosing schedule as part of combination therapy might help increase efficacy, while reducing the side effects of both the ADC and chemotherapy. Certain classes of chemotherapies may also work in a synergistic manner with specific ADC toxins and drugs that increase antigen expression might counter resistance to targeted agents. Addition of ADCs to the armamentarium of oncologists has offered novel and more targeted treatment strategies that might enable clinicians to safely extend the lifespan of patients with various cancer diagnoses.
